# Technetium-99 Conjugated with Methylene Diphosphonate Ameliorates Glucocorticoid Induced Osteoporosis by Inhibiting Osteoclastogenesis

**DOI:** 10.1155/2018/7902760

**Published:** 2018-10-14

**Authors:** Lianjie Shi, Ying Ning, Liling Xu, Jianhong Li, Xuewu Zhang

**Affiliations:** ^1^State Key Laboratory of Natural and Biomimetic Drugs, School of Pharmaceutical Sciences, Peking University, Beijing, China; ^2^Department of Rheumatology and Immunology, Peking University International Hospital, Beijing, China; ^3^Department of Rheumatology and Immunology, Peking University People's Hospital, Beijing, China; ^4^Department of Nuclear Medicine, Peking University International Hospital, Beijing, China

## Abstract

Technetium-99 conjugated with methylene diphosphonate (^99^Tc-MDP) is an effective anti-inflammatory drug in treating rheumatoid arthritis (RA) for over 15 years in China. However, as a special form of bisphosphonate, the antiosteoporotic effect of ^99^Tc-MDP is unclear. We systematically investigated the effects of ^99^Tc-MDP on cancellous and cortical bone, respectively, in glucocorticoid induced osteoporosis (GIO) animal models. Forty-eight Sprague-Dawley rats were randomly divided into six groups: blank, negative control, high dose, medium dose, low dose, and positive control groups. After dexamethasone was given to all groups except the blank group to induce osteoporosis, the rats in different groups were treated with saline, MDP, or different doses of ^99^Tc-MDP. After treatment, all rats were sacrificed, and their tibiae and femora were analyzed with microcomputed tomography (micro-CT), histology and biomechanics. Micro-CT analyses showed that (1) ^99^Tc-MDP reversed glucocorticoid induced bone microarchitecture destruction by increasing BV/TV, Tb.Th, and Tb.N and decreasing BS/BV, Tb.Sp, and TBPf; (2) effect of ^99^Tc-MDP increased as its dosage increased; and (3) ^99^Tc-MDP could improve cortical bone thickness while MDP failed to do so. Micro-CT spatial structure analysis and histology also yielded consistent results, indicating that ^99^Tc-MDP increased trabecular number and connectivity morphologically. Secondly, biomechanics revealed that ^99^Tc-MDP can enhance the extrinsic stiffness of bone by changing bone geometry. Finally, ^99^Tc-MDP could inhibit osteoclastogenesis in PBMCs in human. In conclusion, ^99^Tc-MDP exerted antiosteoporotic effect by improving both cancellous and cortical bone, as well as increasing extrinsic bone stiffness which might be attributed to the its inhibition of osteoclast differentiation. The antiosteoporotic effect of ^99^Tc-MDP may suggest a potential clinical application for patients with GIO.

## 1. Introduction

Glucocorticoid (GC), with its fast and prominent anti-inflammatory and immunosuppressive effects, has been widely used in the treatment of immune-mediated diseases including rheumatoid arthritis (RA) [1]. GC therapy, even in low dose, can lead to significantly decreased radiographic progression of rheumatoid arthritis [2]. However, it can cause significant adverse effects such as glucocorticoid induced osteoporosis (GIO), a most common form of secondary osteoporosis [3, 4]. Therefore, early intervention and management of GIO is crucial for patients receiving GC therapy [5].

While different medications have been developed to address GIO, previous researches have proven that bisphosphonate is particularly effective in preventing and treating glucocorticoid induced bone loss [6–8]. A specific form of bisphosphonate, technetium-99 conjugated with methylene diphosphonate (^99^Tc-MDP), is the radioactive-safe decay product of ^99^mTc-MDP which has no radioactivity and is harmless to human body in long term clinical treatment. It was patented in 1995 (patent No. ZL94113006.1) and approved by the State Food and Drug Administration (SFDA) of China in 2002 (Approval No. H20000218) to treat RA due to its anti-inflammation efficacy.

Study in collagen induced arthritis (CIA) rats has shown that ^99^Tc-MDP is effective in suppressing the production TNF-*α* and IL-1*β* [9]. A very recent study has also shown the serum levels of TNF-*α* and IL-6 are reduced significantly while the level of anti-inflammatory cytokine TGF-*β* was increased in patients with RA who received ^99^Tc-MDP therapy [10]. Interestingly, in their study, they firstly found the anti-inflammation effects of ^99^Tc-MDP might be exerted via upregulating the frequency of CD4+CD25+Foxp3+ Tregs and *γδ* T cell in peripheral blood. Furthermore, a previous study demonstrated that ^99^Tc-MDP decreased the rheumatoid factor concentration in RA patients which indicated ^99^Tc-MDP could also control the immune activities of RA [11].

^99^Tc-MDP has been used in Chinese RA patients for over 15 years based on its anti-inflammation effects. And till now most studies have focused on this efficacy in RA. However, as a bisphosphonate, less attention has been paid to the antiosteoporotic effect [9, 11–13]. The objective of our present study is to systematically investigate the effect of ^99^Tc-MDP on cancellous and cortical bone, respectively, in vivo by applying microcomputed tomography (micro-CT), histomorphology, and biomechanical test, as well as it on the osteoclast formation in vitro.

## 2. Materials and Methods

### 2.1. Animal Model

Forty-eight three-month-old female Sprague-Dawley rats, weighing 256.96 ± 18.18g, were obtained from Beijing Vital River Laboratory Animal Technology Co. Ltd. The rats were kept in the same room of the laboratory animal center of Peking University Health Science Centre, fed on the same diet daily, and weighed weekly. The forty-eight rats were randomly and evenly divided into six groups: blank group, negative control group (saline), high dose group (^99^Tc-MDP 10 mg/kg), medium dose group (^99^Tc-MDP 5 mg/kg), low dose group (^99^Tc-MDP 2.5 mg/kg), and positive control group (MDP 5 mg/kg).

During the induction period, physiological saline was given to the blank group and dexamethasone to the other five groups, 5 mg/kg, i.m, twice a week, for 16 weeks. In the treatment period, the blank and the negative control groups were given 5 mg/kg saline; 10 mg/kg, 5 mg/kg, and 2.5 mg/kg ^99^Tc-MDP were given to the high dose, medium dose, and low dose groups, respectively; and the positive control group was given 5 mg/kg MDP, all intravenously, once a week, for 14 weeks. Dexamethasone was given in the same way mentioned above during the treatment period to maintain osteoporotic effect, except that the frequency was reduced to once a week.

Sterile injection of ^99^Tc-MDP was prepared before use by mixing two preparations. Preparation A was transparent solution containing 0.05 *μ*g 99Tc. Preparation B was lyophilized powder containing 5 mg MDP and 0.5 mg stannous chloride. Both preparations were sealed in 5 mL glass vials and stored in darkness at 2–8°C.

The rats were sacrificed after treatment. Their femora and tibiae were collected for micro-CT analysis, histology, and biomechanical test as described in the following sections. All animal treatments and procedures were approved by the ethical committee of Peking University Health Science Center.

### 2.2. Microcomputed Tomography

#### 2.2.1. Microstructural Quantitative Analysis

The right tibiae were thawed in room temperature; the proximal part was scanned by micro-CT (Siemens Inveon CT/PET, Germany) in high resolution set by Inveon Acquisition Workplace. The voltage was 60 kV and the current was 400 *μ*A; the total rotation was 360 degrees at a rotation step of 2 degrees (integration time was 12 minutes). The effective pixel size was 10.34 *μ*m. The acquired scan data sets were reconstructed at a voxel size of 5.17 × 5.17 × 10.34 *μ*m^3^.

A 2 mm thick region, which was 1 mm distal to the proximal tibial growth plate, was defined as a volume of interest (VOI) for quantitative analysis with Inveon Research Workplace. Six parameters of cancellous bone were also calculated by the workstation: bone volume/total volume (BV/TV), bone surface area/bone volume (BS/BV), trabecular thickness (Tb.Th), trabecular number (Tb.N), trabecular spacing (Tb.Sp), and trabecular bone pattern factor (TBPf). One parameter of cortical bone was calculated: cortical thickness (Ct.Th) by measurements at 8 different sites.

#### 2.2.2. Spatial Structure of the Cancellous Bone

A 0.4 mm thick region, which was 1.8 mm distal to the proximal tibial growth plate, was defined as a VOI for 3D construction to show the spatial structure of cancellous bone.

### 2.3. Histology

The left tibiae were fixed with 4% paraformaldehyde, embedded in paraffin to make 4-*μ*m-thick sections, then stained with hematoxylin and eosin (H & E), and observed under light microscope.

### 2.4. Biomechanical Tests

#### 2.4.1. Extrinsic Biomechanical Properties

Right femora were thawed before three-point bending test and submerged in a saline bath maintained at 37°C. Load was applied midway between a 15-mm loading span at a speed of 6 mm/s using mechanical testing machine (MTS Systems Corp., Eden Prairie, MN, USA). Fu (ultimate force), Du (ultimate displacement), S (stiffness), and U (energy absorbed) were determined from the load-displacement curve recorded by software [14].

### 2.5. Bone Geometry

Bone geometry measurements of the midshaft of the fractured femora were performed using digital calipers (accuracy: 0.005 mm) after three-point bending test, including width of the bone in the mediolateral direction, (a) width of the bone in the anteroposterior direction (b) and cortical thickness (Ct.Th). Average cortical thickness was calculated from thickness measurements made in each of the four directions of the femoral cross-section.

The value for the cross-sectional moment of inertia (CSMI) at the midshaft of the femur was calculated using the elliptical cross-section model [15]:

(1) CSMI=π64ab3−a−2tb−2t3t=Ct.Th

#### 2.5.1. Intrinsic Biomechanical Properties

Intrinsic biomechanical properties were calculated from the following formulas:

(2) σu=FuLb8Iεu=du6bL2E=SL348IμT=U3b24LI

where *σ*_u_ is ultimate stress, *ε*_u_ is ultimate strain, E is elastic modulus, *μ*_*T*_ is modulus of toughness, L is the loading span (15 mm), and I is the CSMI [16].

### 2.6. Osteoclast Differentiation Assay

A total of 7 rheumatoid arthritis (RA) patient peripheral blood mononuclear cells (PBMCs) were isolated from fresh heparinized venous blood samples using Ficoll density-gradient centrifugation and then were plated in a-MEM medium (Life Technologies, Grand Island, NY, USA) supplemented with 10% FBS (Life Technologies) at 5× 10^4^ cells per 200 *μ*l per well in 96-well plates. After adhering for 4 h in the incubator, nonadherent cells were removed to get the adherent osteoclast precursors (>90% CD14+) under the stimulation of 30 ng/ml recombinant human macrophage colony stimulating factor (rhM-CSF, Peprotech GmbH, Rocky Hill, CT, USA)) and 50 ng/ml recombinant human receptor activator of nuclear factor-kappa B ligand (rhRANKL, R&D systems, Minneapolis, MN, USA) with or without 100 ng/ml rhTyro3 Fc chimera. Medium was changed every 3 days. On day 17, the cells were detected by tartrate-resistant acid phosphatase (TRAP) staining with the Leukocyte Acid Phosphatase kit (Sigma-Aldrich, St. Louis, MO, USA) and TRAP positive multinucleated cells (three or more nuclei) were counted under an inverted fluorescence microscope (Olympus IX71-141, Tokyo, Japan).

### 2.7. Statistical Analysis

All statistical analyses were performed using SPSS 17.0. Data were tested for normality and homogeneity of variances. Results were expressed as $\bar{x}\pm s$ and tested for significance using independent or paired* t*-test. Simple linear regression and Pearson's correlation coefficient were calculated to test for linear correlations. P value small than 0.05 was considered statistically significant.

## 3. Results

### 3.1. ^99^Tc-MDP Improved BV/TV, Tb.N, BS/BV, Tb.Sp, and TBPf Significantly in Cancellous Bone, as well as Ct.Th in Cortical Bone in the Rats with GIO

**Figure 1:**
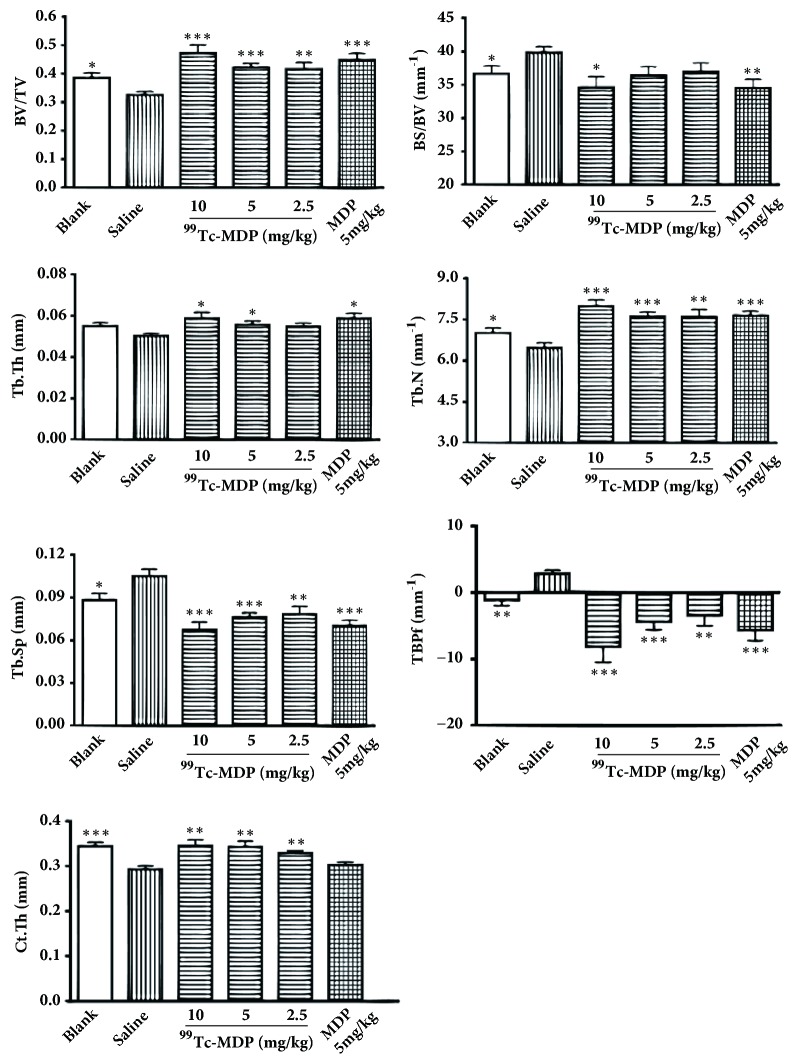
^99^ **Tc-MDP improved microstructural quantitative parameters of cancellous and cortical bone**. Dexamethasone decreased BV/TV and Tb.N and increased BS/BV, Tb.Sp, and TBPf accordingly. However, ^99^Tc-MDP exhibited excellent effects on conversing the parameters in different dose groups. Data are presented as mean ± standard deviation (n = 8, each group). BV/TV: bone volume/total volume; BS/BV: bone surface area/bone volume; Tb.Th: trabecular thickness; Tb.N: trabecular number; Tb.Sp: trabecular spacing; TBPf: trabecular bone pattern factor; and Ct.Th: cortical thickness. All the other five groups are compared with negative control group (saline group), respectively. *∗* p < 0.05, *∗∗* p < 0.01, and *∗∗∗* p < 0.001.

Microstructural quantitative analysis of cancellous bone by microcomputed tomography showed that, (1) compared with the blank group, rats in the negative control group showed significant decreases in BV/TV and Tb.N as well as significant increases in BS/BV, Tb.Sp, and TBPf (p < 0.05); (2) compared with the negative control group, rats in the low dose group showed significant increases in BV/TV and Tb.N and significant decreases in Tb.Sp and TBPf (p < 0.05); (3) for the medium dose and high dose groups, the parameters of Tb.Th and BS/BV showed significantly improvement as the dosing increased; and (4) for the positive control group, improvement of all six parameters reached statistical significance (Table 1 and Figure 1).

Quantitative analysis of cortical bone revealed that (1) Ct.Th decreased in the negative control group in contrast with the blank group (p < 0.05); (2) after treatment, Ct.Th in three ^99^Tc-MDP treatment groups, regardless of dose differences, all increased significantly, comparing with the negative control group (p < 0.05); (3) no significant improvement of Ct.Th was observed in the positive control group, when compared with the negative control group (Table 1 and Figure 1).

### 3.2. ^99^Tc-MDP Ameliorated the Disorder in Structure of Cancellous Induced by Glucocorticoid

**Figure 2:**
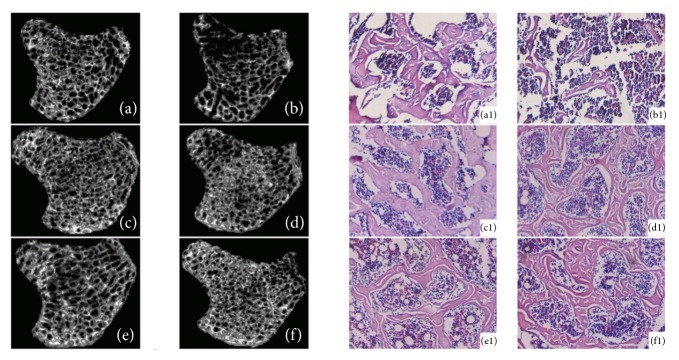
**3D structure of cancellous bone from micro-CT (0.4mm thick) and characteristics of trabeculae visualized by H&E staining (magnification: 50×).**  ^99^Tc-MDP modulated the density, integrity, and connectivity of trabeculae changed by glucocorticoid (left panel). Staining with hematoxylin and eosin (H&E), sparse, and disordered trabeculae induced by glucocorticoid was improved after the treatment of ^99^Tc-MDP (right panel). (a) Blank group, (b) negative control group, (c) high dose group, (d) medium dose group, (e) low dose group, and (f) positive control group.

Structures of cancellous from all the groups were evaluated by 3D structure in the present study. The results showed that, compared with the blank group, trabeculae in the negative control group became thinner and sparse, and the microstructure was compromised. The density, integrity, and connectivity of trabeculae in high dose group, medium dose group, low dose group, and positive control group all improved substantially compared with the negative control group (Figure 2, left panel).

H&E staining was also performed in our present study. Our finding showed that trabeculae in the negative control group became sparse and disordered compared with the blank group; by contrast, trabeculae in high dose group, medium dose group, low dose group, and positive control group all became denser and regular, which was consistent with the 3D structure mentioned above (Figure 2, right panel).

### 3.3. The Efficacy of ^99^Tc-MDP on Stiffness, Cortical Thickness, and Cross-Sectional Moment of Inertia

**Figure 3:**
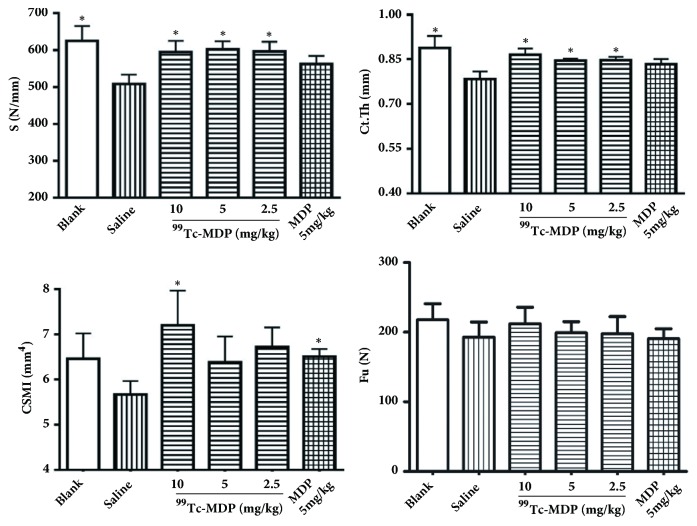
**Bone mechanical properties of femora midshaft from rats were evaluated.** ^99^Tc-MDP increased S (stiffness), Ct.Th (cortical thickness), and CSMI (cross-sectional moment of inertia) at the femoral midshaft of rats. However, ^99^Tc-MDP showed no effects on Fu (ultimate force). All the other five groups are compared with negative control group (group saline), respectively. *∗*p < 0.05.

Compared with the blank group, stiffness (S), cortical thickness (Ct.Th), and cross-sectional moment of inertia (CSMI) all decreased in the negative control group. However, only the decreases in S and Ct.Th were statistically significant. In addition, all the three ^99^Tc-MDP treatment groups, when compared with the negative control group, showed increases in S and Ct.Th (P < 0.05), but only the high dose group showed significant increase in CSMI. S and Ct.Th increased in the positive control group, but there was no statistical significance. The positive control group showed significant increase in CSMI (p < 0.05) (Table 2 and Figure 3).

However, ultimate force (Fu), energy absorbed (U), elastic modulus (E), and modulus of toughness (*υ*T) did not vary significantly between the groups (Table 2).

### 3.4. ^99^Tc-MDP Inhibited Osteoclastogenesis in Human PBMCs In Vitro

**Figure 4:**
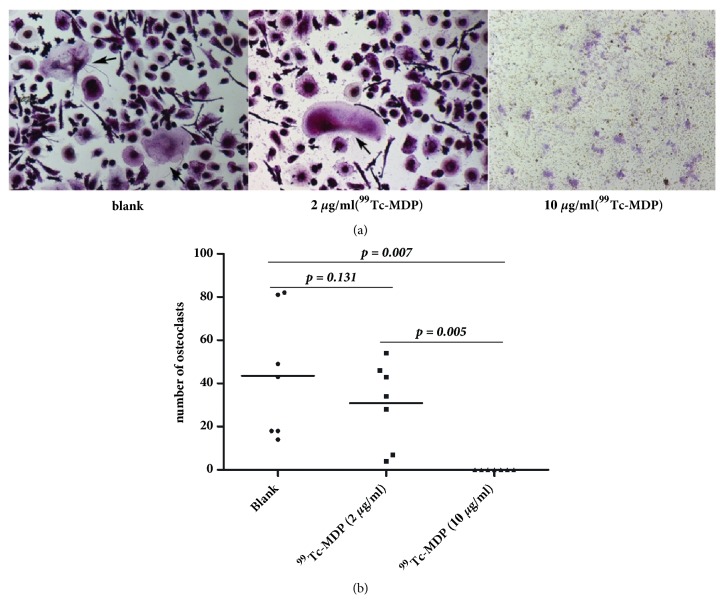
**Osteoclastogenesis in human PBMCs could be inhibited by** ^**99**^ **Tc-MDP**. (a) Osteoclast number in per well was counted under an inverted fluorescence microscope. Images collected at 400× magnification. (b) The differences between every two groups were evaluated by independent *t* test. A p < 0.05 was considered to be statistically significant.

To further investigate the underlying mechanism of ^99^Tc-MDP on GIO. We evaluated the osteoclast inhibition effect of ^99^Tc-MDP in human PBMCs in vitro by TRAP staining. Seven RA patients were enrolled in our present study. PBMCs were isolated and stimulated with RANKL and M-CSF to promote macrophage differentiated to osteoclasts. All groups except the blank group were treated with 2 *μ*g/ml or 10 *μ*g/ml ^99^Tc-MDP, respectively. ^99^Tc-MDP with a concentration of 2 *μ*g/ml showed no significant inhibition, but with 10 *μ*g/ml exhibited complete inhibition on osteoclastogenesis (Figures 4(a) and 4(b)).

## 4. Discussion

In the present study, as we known, we are the first to show that ^99^Tc-MDP ameliorates glucocorticoid induced osteoporosis in vivo. We demonstrate the anti-GIO effects of ^99^Tc-MDP by microcomputed tomography, bone histomorphology, and biomechanics in vivo and antiosteoporosis by TRAP staining in vitro. Our findings indicate that ^99^Tc-MDP effectively ameliorates glucocorticoid induced osteoporosis by inhibiting osteoclastogenesis.

Micro-CT, as our major investigation tool to quantify bone microstructure, was able to deliver detailed and accurate anatomical information and measurements, due to its ability to achieve high spatial resolution [17, 18]. It was first introduced by Feldkamp, L.A., to directly examine three-dimensional bone structure in vitro; structural indices commonly determined from two-dimensional histological sections can be obtained nondestructively from a large number of slices in each of three orthogonal directions with little or no preparation of the sample [18–20]. Since then, this technology has become a powerful tool for assessing the 3D architecture of trabecular bone. Measurements from micro-CT were excellently correlated with those measured from conventional histomorphometry and therefore were very reliable [18, 21–25].

Our study had three important findings. First, low dose of ^99^Tc-MDP (2.5 mg/kg/week) was able to significantly reverse the microstructural change of cancellous and cortical bone caused by long term glucocorticoid use. Our research is the first one to confirm that ^99^Tc-MDP may not only improve cancellous bone, demonstrated by increased BV/TV and Tb.N and decreased Tb.Sp and TBPf, but also thicken cortical thickness impaired by glucocorticoid. Second, besides quantitative evidence described above, morphological evidence derived from both micro-CT 3D structure and histology of cancellous bone consistently showed that ^99^Tc-MDP treatment yielded denser, thickened trabeculae, and more integrated cancellous structure compared with the negative control group. Third, ^99^Tc-MDP demonstrated capability to change bone geometry such as increasing cortical thickness and CSMI, therefore to enhance bone extrinsic stiffness in biomechanical test. There was no significant intrinsic stiffness change after ^99^Tc-MDP treatment.

By comparing effects of ^99^Tc-MDP and MDP, we found that MDP also showed excellent ability to improve cancellous bone. However, it has no effect on cortical bone thickness (as shown in both micro-CT measurements and digital caliper measurements) and bone extrinsic stiffness impaired by glucocorticoid.

As for the underlying antiosteoporotic mechanism, the P-C-P structure of MDP enables ^99^Tc-MDP to localize in bone and joint after injection, then inhibits activity of osteoclasts and bone absorption and promotes osteoblastogenesis and bone formation, which may be one of the most important antiosteoporotic mechanism of ^99^Tc-MDP. In addition, microelement ^99^Tc has a long half-life in human body and therefore affords ^99^Tc-MDP to exert its therapeutic effects in a long term; this characteristic is distinct from conventional drug relying on plasma concentration to play a role and is probably the reason why ^99^Tc-MDP appears to be more effective than MDP on improving cortical bone.

To better understand the antiosteoporotic mechanism of ^99^Tc-MDP, we performed the in vitro study in PBMCs from RA patients. We found ^99^Tc-MDP could inhibit M-SCF and RANKL induced osteoclastogenesis which was consistent with a recent study of effect of ^99^Tc-MDP on ovariectomy-induced osteoporotic in mice [13]. In their study, they also found the ^99^Tc-MDP could upregulate CD4+CD25+Foxp3+ Tregs and downregulate Th17 cells in spleen in ovariectomy-induced osteoporotic mice. The upregulation of CD4+CD25+Foxp3+ Tregs by ^99^Tc-MDP was also found by Wu and her colleagues in peripheral blood from RA patients [12]. Were CD4+CD25+Foxp3+ Tregs involved in the development of osteoporosis? Tregs were hypothesized to exert the inhibitory effects on osteoclast formation in several studies [26–28]. It suggested that the inhibition of osteoclast formation by ^99^Tc-MDP might be a result of the upregulation of Tregs.

In conclusion, the data presented here provide new insight into the underlying effects of ^99^Tc-MDP in improving both cancellous and cortical bone, and increasing extrinsic bone stiffness might be via inhibiting osteoclastogenesis. However, the current data are insufficient to determine whether the antiosteoporotic effect of ^99^Tc-MDP, in patients with GIO, is as effective as it in vivo and in vitro. Hence, ongoing studies have been designed to assure the antiosteoporotic effects of ^99^Tc-MDP in patients with GIO.

## Figures and Tables

**Table 1:** Microstructural quantitative analysis of cancellous and cortical bone in six groups

Parameters	Blank	Negative control	High Dose	Medium Dose	Low Does	Positive Control
BV/TV	0.386 ± 0.048^*∗*^	0.326 ± 0.032	0.471 ± 0.086^&^	0.421 ± 0.042^&^	0.416 ± 0.065^#^	0.449 ± 0.064^&^
BS/BV (mm^−1^)	36.64 ± 3.08^*∗*^	39.84 ± 2.29	34.56 ± 4.71^*∗*^	36.37 ± 3.78	39.93 ± 3.81	34.49 ± 3.78^#^
Tb.Th (mm)	0.0549 ± 0.0048	0.0503 ± 0.0029	0.0588 ± 0.0079^*∗*^	0.0555 ± 0.0057^*∗*^	0.0546 ± 0.0053	0.0587 ± 0.0071^*∗*^
Tb.N (mm^−1^)	7.008 ± 0.468^*∗*^	6.466 ± 0.480	7.980 ± 0.669^&^	7.607 ± 0.469^&^	7.591 ± 0.784^#^	7.640 ± 0.452^&^
Tb.Sp (mm)	0.0883 ± 0.0123^*∗*^	0.1051 ± 0.0131	0.0673 ± 0.0156^&^	0.0764 ± 0.0085^&^	0.0783 ± 0.0160^*∗∗*^	0.0702 ± 0.0117^&^
TBPf (mm^−1^)	-1.08 ± 2.36^#^	2.87 ± 1.36	-8.10 ± 6.65^&^	-4.33 ± 3.54^&^	-3.37 ± 4.52^#^	-5.62 ± 4.50^&^
Ct.Th (mm)	0.344 ± 0.0237^&^	0.293 ± 0.0206	0.345 ± 0.0382^#^	0.343 ± 0.0365^#^	0.329 ± 0.0155^#^	0.303 ± 0.0191

Data are presented as mean ± standard deviation. BV/TV: bone volume/total volume; BS/BV: bone surface area/bone volume; Tb.Th: trabecular thickness; Tb.N: trabecular number; Tb.Sp: trabecular spacing; TBPf: trabecular bone pattern factor; and Ct.Th: cortical thickness. All other five groups are compared with negative control group (group B), respectively. ^*∗*^ p < 0.05. ^#^ p < 0.01. ^&^ p < 0.001.

**Table 2:** Biomechanical properties of femora in six groups.

	Parameters	Blank	Negative control	High Dose	Medium Dose	Low Does	Positive Control
Extrinsic parameters	Fu(N)	217.79 ± 23.11	192.77 ± 21.83	212.09 ± 23.72	199.02 ± 16.80	197.88 ± 24.45	190.54 ± 14.36
	Du (mm)	0.655 ± 0.084	0.856 ± 0.20	0.739 ± 0.099	0.687 ± 0.060	0.728 ± 0.096	0.665 ± 0.059
	S(N/mm)	625.53 ± 99.31^*∗*^	508.87 ± 66.03	595.16 ± 80.14^*∗*^	603.27 ± 60.13^*∗*^	596.87 ± 69.75^*∗*^	562.52 ± 61.74
	U (mJ)	89.57 ± 24.43	87.74 ± 25.17	98.66 ± 25.50	85.20 ± 14.87	89.83 ± 22.20	77.69 ± 10.00

Bone geometry	b (mm)	3.17 ± 0.17	3.13 ± 0.13	3.29 ± 0.21	3.21 ± 0.24	3.23 ± 0.16	3.24 ± 0.08
	a (mm)	4.32 ± 0.16	4.07 ± 0.15	4.32 ± 0.36	4.13 ± 0.20	4.31 ± 0.23	4.21 ± 0.16
	Ct.Th (mm)	0.89 ± 0.10^*∗*^	0.78 ± 0.07	0.87 ± 0.06^*∗*^	0.85 ± 0.02^*∗*^	0.85 ± 0.03^*∗*^	0.83 ± 0.05
	CSMI (mm^4^)	6.47 ± 1.37	5.67 ± 0.80	7.20 ± 2.03^*∗*^	6.38 ± 1.62	6.72 ± 1.14	6.51 ± 0.48^*∗*^

Intrinsic parameters	*σ*_u_ (MPa)	203.09 ± 19.65	200.46 ± 17.76	186.41 ± 18.10	191.72 ± 15.21	179.82 ± 14.61	177.29 ± 15.73
	*ε*_u_ (%)	5.56 ± 0. 95	7.14 ± 1.68	6.50 ± 1.03	5.88 ± 0.67	6.28 ± 0.92	5.64 ± 0.37
	E (GPa)	6.96 ± 1.43	6.42 ± 1.31	6.01 ± 0.99	6.85 ± 1.10	6.32 ± 0.71	6.09 ± 0.67
	*υ*_T_ (MJ/ m^3^)	6.97 ± 1.39	7.53 ± 1.79	7.51 ± 1.45	6.98 ± 1.04	7.04 ± 1.66	6.26 ± 0.81

Data are presented as mean ± standard deviation. Extrinsic parameters: Fu (ultimate force), Du (ultimate displacement), S (stiffness), and U (energy absorbed); bone geometry: b (anterior-posterior width), a (media-lateral width), Ct.Th (cortical thickness), and CSMI (cross-sectional moment of inertia); intrinsic parameters: *σ*_u_ (ultimate stress), *ε*_u_ (ultimate strain), E (elastic modulus), and *υ*_T_ (modulus of toughness). All the other five groups are compared with the negative control group (saline group), respectively. ^*∗*^p < 0.05.

## Data Availability

The data used to support the findings of this study are available from the corresponding author upon request.

## References

[1] Hoes J. N., Jacobs J. W. G., Buttgereit F., Bijlsma J. W. J. (2010). Current view of glucocorticoid co-therapy with DMARDs in rheumatoid arthritis. *Nature Reviews Rheumatology*.

[2] Bijlsma J. W. J. (2012). Disease control with glucocorticoid therapy in rheumatoid arthritis. *Rheumatology*.

[3] Van Der Goes M. C., Jacobs J. W. G., Boers M. (2010). Monitoring adverse events of low-dose glucocorticoid therapy: EULAR recommendations for clinical trials and daily practice. *Annals of the Rheumatic Diseases*.

[4] Canalis E., Mazziotti G., Giustina A., Bilezikian J. P. (2007). Glucocorticoid-induced osteoporosis: pathophysiology and therapy. *Osteoporosis International*.

[5] Compston J. E. (2007). Emerging consensus on prevention and treatment of glucocorticoid-induced osteoporosis. *Current Rheumatology Reports*.

[6] Rizzoli R., Adachi J. D., Cooper C. (2012). Management of glucocorticoid-induced osteoporosis. *Calcified Tissue International*.

[7] Reid D. M., Devogelaer J.-P., Saag K. (2009). Zoledronic acid and risedronate in the prevention and treatment of glucocorticoid-induced osteoporosis (HORIZON): a multicentre, double-blind, double-dummy, randomised controlled trial. *The Lancet*.

[8] Thomas T., Horlait S., Ringe J. D. (2013). Oral bisphosphonates reduce the risk of clinical fractures in glucocorticoid-induced osteoporosis in clinical practice. *Osteoporosis International*.

[9] Wang L., Gu Q., Xu Y. (2008). Effects of Yunke (technetium-99 conjugated with methylene diphosphonate; 99Tc-MDP) and/or colloidal chromic phosphate phosphonium-32, alone and in combination, in rats with adjuvant arthritis. *Clinical and Experimental Pharmacology and Physiology*.

[10] Su D., Shen M., Gu B. (2016). (99) Tc-methylene diphosphonate improves rheumatoid arthritis disease activity by increasing the frequency of peripheral gammadelta T cells and CD4(+) CD25(+) Foxp3(+) Tregs. *International Journal of Rheumatic Diseases*.

[11] Anbin H., Likai Y., Lingxun S. (2003). Effect of technetium-99 conjugated with methylene diphosphonate on IgM-RF, IgG-RF and IgA-RF. *Journal of Huazhong University of Science and Technology (Medical Sciences)*.

[12] Wu Q., Ni Y., Yang Q., Sun H. (2016). 99Tc-MDP treatment for the therapy of rheumatoid arthritis, choroidal neovascularisation and Graves' ophthalmopathy. *Biomedical Reports*.

[13] Zhao Y., Wang L., Liu Y. (2012). Technetium-99 conjugated with methylene diphosphonate ameliorates ovariectomy-induced osteoporotic phenotype without causing osteonecrosis in the jaw. *Calcified Tissue International*.

[14] Turner C. H., Roeder R. K., Wieczorek A., Foroud T., Liu G., Peacock M. (2001). Variability in skeletal mass, structure, and biomechanical properties among inbred strains of rats. *Journal of Bone and Mineral Research*.

[15] Turner C. H., Akhter M. P., Raab D. M., Kimmel D. B., Recker R. R. (1991). A noninvasive, in vivo model for studying strain adaptive bone modeling. *Bone*.

[16] Turner C. H., Burr D. B. (1993). Basic biomechanical measurements of bone: a tutorial. *Bone*.

[17] Du L. Y., Umoh J., Nikolov H. N., Pollmann S. I., Lee T.-Y., Holdsworth D. W. (2007). A quality assurance phantom for the performance evaluation of volumetric micro-CT systems. *Physics in Medicine and Biology*.

[18] Bonse U., Busch F., Günnewig O. (1994). 3D computed X-ray tomography of human cancellous bone at 8 *μ*m spatial and 10−4 energy resolution. *Bone and Mineral*.

[19] Feldkamp L. A., Goldstein S. A., Parfitt A. M., Jesion G., Kleerekoper M. (1989). The direct examination of three-dimensional bone architecture in vitro by computed tomography. *Journal of Bone and Mineral Research*.

[20] Rüegsegger P., Koller B., Müller R. (1996). A microtomographic system for the nondestructive evaluation of bone architecture. *Calcified Tissue International*.

[21] Kuhn J. L., Goldstein S. A., Feldkamp L. A., Goulet R. W., Jesion G. (1990). Evaluation of a microcomputed tomography system to study trabecular bone structure. *Journal of Orthopaedic Research*.

[22] Laib A., Barou O., Vico L., Lafage-Proust M. H., Alexandre C., Rügsegger P. (2000). 3D micro-computed tomography of trabecular and cortical bone architecture with application to a rat model of immobilisation osteoporosis. *Medical & Biological Engineering & Computing*.

[23] Kapadia R. D., Stroup G. B., Badger A. M. (1998). Applications of micro-CT and MR microscopy to study pre-clinical models of osteoporosis and osteoarthritis. *Technology and Health Care*.

[24] Glatt M., Daculsi G. (2001). The bisphosphonate zoledronate prevents vertebral bone loss in mature estrogen-deficient rats as assessed by micro-computed tomography. *European Cells and Materials*.

[25] Thomsen J. S., Laib A., Koller B., Prohaska S., Mosekilde L., Gowin W. (2005). Stereological measures of trabecular bone structure: comparison of 3D micro computed tomography with 2D Histological sections in human proximal tibial bone biopsies. *Journal of Microscopy*.

[26] Talaat R. M., Sidek A., Mosalem A., Kholief A. (2015). Effect of bisphosphonates treatment on cytokine imbalance between TH17 and Treg in osteoporosis. *Inflammopharmacology*.

[27] Sakaguchi S., Ono M., Setoguchi R. (2006). Foxp3^+^CD25^+^CD4^+^ natural regulatory T cells in dominant self-tolerance and autoimmune disease. *Immunological Reviews*.

[28] Brunkow M. E., Jeffery E. W., Hjerrild K. A. (2001). Disruption of a new forkhead/winged-helix protein, scurfin, results in the fatal lymphoproliferative disorder of the scurfy mouse. *Nature Genetics*.

